# Association between the triglyceride–glucose index and all-cause and CVD mortality in the young population with diabetes

**DOI:** 10.1186/s12933-024-02269-0

**Published:** 2024-05-16

**Authors:** Chang Liu, Dan Liang, Kun Xiao, Lixin Xie

**Affiliations:** 1 School of Medicine, Nankai University, Tianjin, China; 2 Department of Endocrine, People’ss Hospital of Chongqing Liang Jiang New Area, Chongqing, China; 3 West China Medical College of Sichuan University, Sichuan, China; 4 College of Pulmonary and Critical Care Medicine, Chinese People’s Liberation Army (PLA) General Hospital, Beijing, China

**Keywords:** TyG index, CVD, Diabetes, Mortality

## Abstract

**Background:**

Although studies have demonstrated the value of the triglyceride–glucose (TyG) index for cardiovascular disease (CVD) and cardiovascular mortality, however, few studies have shown that the TyG index is associated with all-cause or CVD mortality in young patients with diabetes. This study aimed to investigate the association between the TyG index and all-cause and CVD mortality in young patients with diabetes in the United States.

**Methods:**

Our study recruited 2440 young patients with diabetes from the National Health and Nutrition Examination Survey (NHANES) 2001–2018. Mortality outcomes were determined by linking to National Death Index (NDI) records up to December 31, 2019. Cox regression modeling was used to investigate the association between TyG index and mortality in young patients with diabetes. The nonlinear association between TyG index and mortality was analyzed using restricted cubic splines (RCS), and a two-segment Cox proportional risk model was constructed for both sides of the inflection point.

**Results:**

During a median follow-up period of 8.2 years, 332 deaths from all causes and 82 deaths from cardiovascular disease were observed. Based on the RCS, the TyG index was found to have a U-shaped association with all-cause and CVD mortality in young patients with diabetes, with threshold values of 9.18 and 9.16, respectively. When the TyG index was below the threshold value (TyG index < 9.18 in all-cause mortality and < 9.16 in CVD mortality), its association with all-cause and CVD mortality was not significant. When the TyG index was above the threshold (TyG index ≥ 9.18 in all-cause mortality and ≥ 9.16 in CVD mortality), it showed a significant positive association with all-cause mortality and CVD mortality (HR 1.77, 95% CI 1.05–2.96 for all-cause mortality and HR 2.38, 95% CI 1.05–5.38 for CVD mortality).

**Conclusion:**

Our results suggest a U-shaped association between TyG index and all-cause and CVD mortality among young patients with diabetes in the United States, with threshold values of 9.18 and 9.16 for CVD and all-cause mortality, respectively.

## Introduction

The incidence and mortality rates of cardiovascular diseases (CVDs) are on a steady rise, presenting a significant threat to both the global economy and human health. This surge in CVDs has emerged as a major public health challenge of global significance [1, 2]. Recent reports indicate a staggering increase in the prevalence of cardiovascular diseases worldwide, with cases soaring from 271 to 523 million over the past three decades [3]. Additionally, the number of cardiovascular-related deaths has surged by 6.5 million cases during this period [3]. Consequently, early identification of individuals with high cardiovascular risk factors is imperative to mitigate the prevalence and mortality rates associated with these conditions.

Insulin resistance is a state of pathophysiological impairment characterized by reduced sensitivity and response of the body to insulin, which ultimately leads to hyperglycemia [4, 5]. The hyperinsulin–hyperglycemic clamp is the gold standard for the assessment of IR, and its complexity leads to limited value in clinical application [6]. The homeostasis model assessment of insulin resistance (HOMA-IR), which uses fasting glucose and insulin levels indirectly to assess the degree of IR, is also limited in its widespread clinical use because of the high insulin measurement requirements and poor reproducibility [7]. In recent years, the triglyceride–glucose product index (TyG index), calculated from triglyceride and fasting glucose levels, has emerged as a potentially simple, convenient, and cost-effective alternative for detecting IR [8, 9]. Notably, the TyG index demonstrates superior predictability of IR compared to HOMA-IR, with an area under the curve (AUC) of 0.784 for the TyG index versus 0.728 for HOMA-IR [10]. The TyG index has demonstrated its capability to predict adverse clinical outcomes in patients with cardiovascular disease (CVD) [11–13]. Moreover, it is intricately linked to the risk of developing carotid atherosclerosis [14], experiencing premature stroke [15], and encountering acute decompensated heart failure [16]. Notably, the prevalence of CVD among individuals with type 2 diabetes can be as high as 32.2% [17]. Given its role as an indicator of insulin resistance, the TyG index exhibits a close association with type 2 diabetes as well [18].

Given the limited scope of existing studies on the association between the TyG index and all-cause mortality and CVD mortality in young patients with diabetes, further validation is warranted. Therefore, this study aimed to provide epidemiologic evidence to demonstrate that the TyG index is an important marker of all-cause mortality and CVD mortality in young patients with diabetes.

## Materials and methods

National Health and Nutrition Examination Survey (NHANES), a continuous survey, employs complex multistage probability sampling to select a representative group of Americans, assessing the health and nutritional status of both adults and children. The Centers for Disease Control and Prevention (CDC) is tasked with providing health statistics for the nation. The NHANES study plan received approval from the Ethics Review Committee of the National Center for Health Statistics (NCHS). All study participants provided written informed consent. The datasets generated and analyzed in this study are readily accessible on the website (https://www.cdc.gov/nchs/nhanes/index.html).

### Study population

**Fig. 1:**
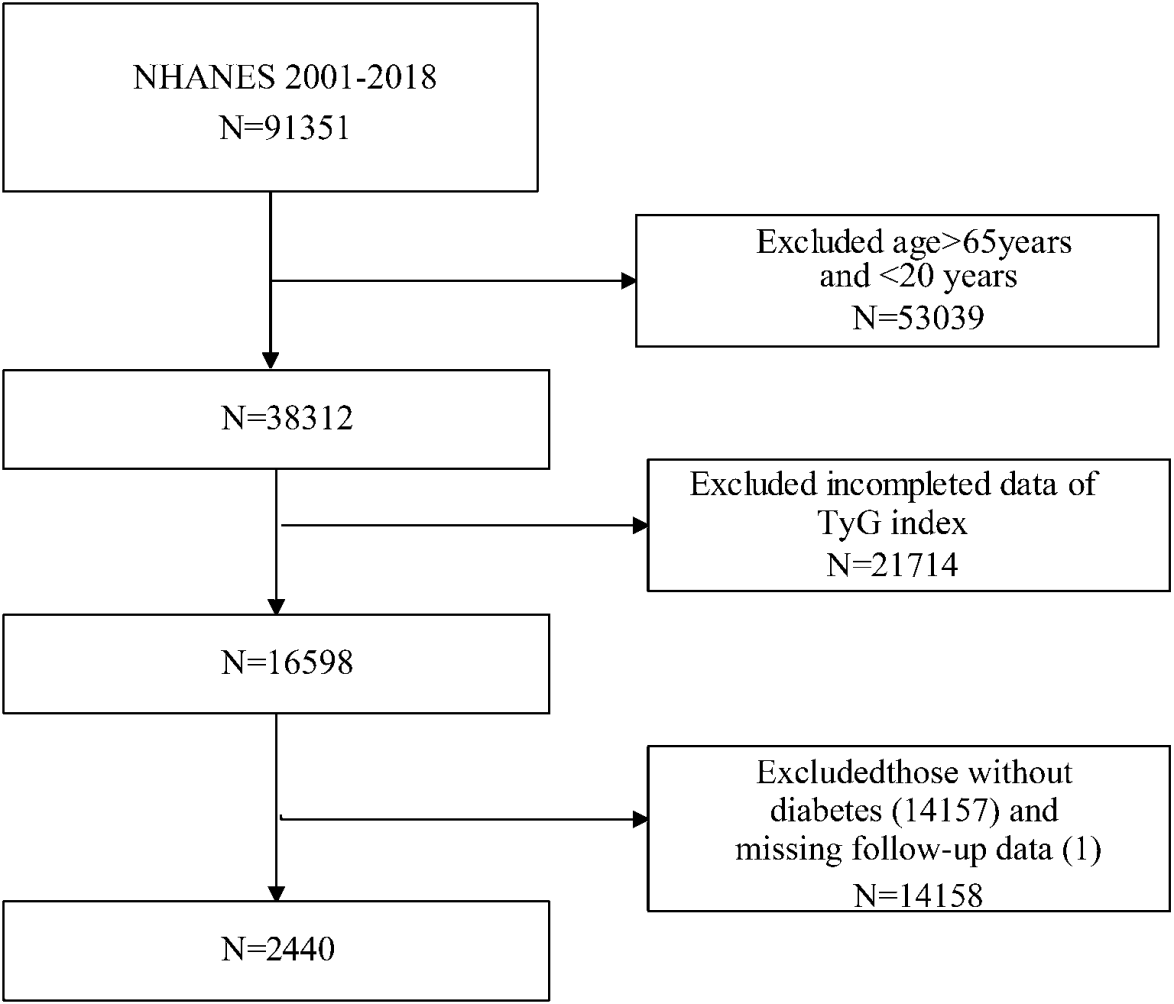
Flowchart of the sample selection from National Health and Nutrition Examination Survey (NHANES) 2001–2008

The data analyzed in this study spanned from the 2001 to 2018 NHANES database. Initially, our study enrolled 91,351 participants. Following the exclusion of individuals over 65 years of age and under 20 years of old (N = 53,039), and those with missing data on the TyG index (N = 21,714) and the follow-up data (N = 1), and those without diabetes (N = 14,157), the final analysis included 2440 participants (Fig. 1).

### Assessment of TyG index

TyG served as the chosen exposure variable, and we calculated the TyG index using the formula Ln [triglycerides (mg/dl) * fasting glucose (mg/dl)/2]. The concentrations of triglycerides and fasting glucose underwent determination through an enzymatic assay performed on an automatic biochemistry analyzer. Specifically, the Roche Modular P and Roche Cobas 6000 chemistry analyzers were employed to measure serum triglyceride concentration. Additionally, fasting plasma glucose levels were evaluated through the hexokinase-mediated reaction, utilizing the Roche/Hitachi Cobas C 501 chemistry analyzer.

### Assessment of mortality

To determine the mortality status in the follow-up population, we utilized the NHANES public-use linked mortality file, updated as of December 31, 2019. This file underwent linkage with the National Death Index (NDI) by the NCHS using a probability matching algorithm. Additionally, disease-specific deaths were identified using the International Statistical Classification of Diseases, 10th Revision (ICD-10). Cardiovascular deaths (including rheumatic heart disease, hypertensive heart disease, ischemic heart disease, acute myocardial infarction, pericardial disease, and acute myocarditis and heart failure) correspond to disease codes I00–I09, I11, I13, and I20–I51.

### Assessment of covariates

Data on diverse demographic and health-related factors were collected through NHANES household interviews. This comprehensive information included details such as age, gender (female/male), race/ethnicity (Mexican American/Non-Hispanic Black/Non-Hispanic White/Others), education levels (less than 9th grade/9–11th grade/high school graduate/some college or AA degree/college graduate or above), the ratio of family income to poverty (< 1/1–4/ > 4), alcohol use (yes/no), smoking status (never/former/now), and disease status (diabetes/hypertension). Diabetes (DM) was defined as either treatment or medical diagnosis of hyperglycemia with hemoglobin A1c ≥ 6.5%, fasting blood glucose ≥ 126 mg/dl, or a 2-h blood glucose ≥ 200 mg/dl [19]. Hypertension was defined as the use of antihypertensive medications, a medical diagnosis of hypertension, or three consecutive measurements of systolic blood pressure ≥ 140 mmHg or diastolic blood pressure ≥ 90 mmHg [20]. BMI was computed by dividing weight (in kilograms) by the square of height (in meters). Participants were classified as normal weight (< 25 kg/m^2^), overweight (25–29.9 kg/m^2^), or obese (≥ 30 kg/m^2^) based on their BMI. Total cholesterol (TC), triglyceride (TG), fasting glucose, high-density lipoprotein cholesterol (HDL-C), low-density lipoprotein cholesterol (LDL-C), and glycosylated hemoglobin (HbA1c) were measured in the laboratory.

### Statistical analysis

Statistical analyses adhered to the guidelines outlined by the CDC. Considering the intricate probability sample design and deliberate oversampling of specific populations in NHANES to ensure representativeness, sample weights were applied to amalgamate data from multiple survey cycles. Participants in the study were categorized into four groups based on quartiles (Q1–Q4) of the TyG index. Continuous variables were expressed as means ± standard deviations, while categorical variables were represented as percentages and corresponding 95% confidence intervals (CI). The weighted one-way ANOVA was employed for continuous variables, and the weighted chi-square test was utilized for categorical variables to evaluate differences in the descriptive analyses. To evaluate the hazard ratios (HR) and 95% CI for the association between the TyG index and the risk of all-cause mortality and CVD mortality, multivariate Cox proportional hazards regression models were developed. There were three models to control for confounding factors. Model 1 was unadjusted, Model 2 was adjusted for age, gender, and race. Model 3 was adjusted for age, gender, race, educational levels, PIR, BMI, hypertension, HDL-C, LDL-C, alcohol use, and smoking status. Multiple imputation was performed for covariates with missing values. The dose–response association between the TyG index and mortality was explored using restricted cubic spline (RCS) analysis. If the association exhibited nonlinearity, the threshold value was estimated by trying all possible values, choosing the threshold point with the highest likelihood. Subsequently, a two-piecewise Cox proportional risk model was employed on both sides of the inflection point to investigate the association between the TyG index and the risk of all-cause mortality and CVD mortality. For subgroup analysis of the association between the TyG index and the risk of all-cause mortality and CVD mortality, the data were stratified by gender (male/female), BMI (normal weight/overweight/obesity), hypertension (yes/no), smoking status (never/former/now) and alcohol use (yes/no). These stratified factors were also considered as potential effect modifiers. A significance level of two-tailed *P* < 0.05 was used to indicate statistical significance. All analyses were conducted using R version 4.3.2 (http://www.R-project.org, The R Foundation).

## Results

### Baseline characteristics of study participants

Table 1 presented the baseline characteristics of the study participants, stratified by quartiles of the TyG index. In total, 2440 participants were enrolled in our study, with an average age of 51.14 ± 0.28 years. Among them, 47.08% were female, and 52.92% were male. In addition, among these participants, 8.80% had no high school or higher education, 15.49% had poor household income, 63.13% were obese, 62.86% had hypertension, 72.54% were alcohol users, and 23.44% were current smokers. The average TyG index in the recruited subjects was 9.27 ± 0.02, and the TyG index range for quartiles 1–4 were 5.64–8.75, 8.75–9.18, 9.18–9.72, and 9.72–13.40, respectively. Statistically significant differences were in total cholesterol, triglycerides, fasting glucose, HDL-C, LDL-C, HbA1c%, BMI, alcohol consumption, and hypertension across the TyG index quartiles (all *P* < 0.05). Compared with the lowest TyG index quartile, individuals with increased TyG index group were more likely to have hypertension, elevated levels of total cholesterol, triglycerides, fasting glucose, LDL-C, HbA1c%, and BMI. Additionally, they exhibited a greater likelihood of being Mexican American and non-alcohol users. There was no statistically significant difference among quartiles in age, gender, educational levels, PIR, and smoking status (all *P* > 0.05).

**Table 1:** Weighted baseline characteristics of the study population

TyG index	All participants	Quartile 1 (5.64–8.75)	Quartile 2 (8.75–9.18)	Quartile 3 (9.18–9.72)	Quartile 4 (9.72–13.40)	*P* value
Age (year)	51.14 (0.28)	50.52 (0.56)	51.86 (0.48)	51.84 (0.50)	50.18 (0.60)	0.05
Total cholesterol (mg/dl)	33.76 (0.25)	171.77 (2.38)	187.94 (2.10)	194.65 (2.23)	218.68 (3.15)	** < 0.0001**
Triglyceride (mg/dl)	184.35 (5.87)	77.00 (1.48)	125.49 (2.01)	178.25 (2.24)	366.20 (19.93)	** < 0.0001**
Fast glucose (mg/dl)	156.52 (1.62)	117.91 (1.63)	136.16 (2.01)	149.58 (1.82)	226.16 (3.93)	** < 0.0001**
HDL-C (mg/dl)	47.30 (0.45)	54.93 (0.78)	49.01 (0.68)	44.39 (0.51)	40.85 (0.63)	** < 0.0001**
LDL-C (mg/dl)	111.49 (1.10)	101.32 (2.03)	113.70 (1.89)	114.81 (2.13)	116.83 (2.67)	** < 0.0001**
HbA1c (%)	7.16 (0.05)	6.33 (0.05)	6.66 (0.08)	6.96 (0.07)	8.78 (0.09)	** < 0.0001**
BMI (kg/m^2^)	33.76 (0.25)	32.84 (0.49)	33.71 (0.48)	33.95 (0.36)	34.50 (0.46)	** < 0.0001**
TyG index	9.27 (0.02)	8.34 (0.02)	8.98 (0.01)	9.43 (0.01)	10.36 (0.03)	** < 0.0001**
Gender, %						0.08
Female	47.08 (42.93,51.22)	47.92 (42.69, 53.15)	50.57 (44.15, 57.00)	48.29 (43.26, 53.3)	40.91 (36.03, 45.79)	
Male	52.92 (48.01,57.84)	52.08 (46.85, 57.31)	49.43 (43.00, 55.85)	51.71 (46.68, 56.7)	59.09 (54.21, 63.97)	
Races, %						** < 0.0001**
Mexican American	11.36 (9.54,13.19)	7.36 (5.42, 9.30)	9.65 (7.33,11.8)	12.72 (9.34, 16.11)	15.87 (12.56, 19.19)	
Non-hispanic black	14.95 (13.12,16.78)	24.75 (20.83, 28.66)	13.68 (10.69, 16.67)	11.29 (8.75, 13.83)	10.50 ( 7.87, 13.13)	
Non-hispanic white	57.82 (51.63,64.42)	52.61 (47.56, 57.67)	61.21 (55.72, 66.71)	62.27 (56.59, 67.95)	54.36 (48.93, 59.80)	
Others	15.87 (13.47,18.26)	15.28 (12.09, 18.48)	15.45 (11.77, 19.13)	13.71 (10.32, 17.11)	19.26 (15.15, 23.36)	
Educational levels, %						0.14
Less than 9th grade	8.80 (7.41, 10.18)	5.29 (3.63, 6.94)	8.62 (6.10, 11.13)	10.17 (7.73, 12.60)	11.05 (8.26, 13.84)	
9–11th grade	13.29 (11.37,15.20)	12.29 ( 9.10, 15.48)	12.00 (8.42, 15.58)	12.93 (9.71, 16.15)	16.15 (12.20, 20.10)	
High school graduate	26.91 (23.26,30.56)	26.89 (21.78, 31.99)	26.58 (21.21, 31.95)	27.76 (22.63, 32.90)	26.38 (21.89, 30.88)	
Some college or AA degree	31.31 (27.75,34.50)	32.87 (27.00, 38.73)	30.03 (24.97, 35.09)	30.51 (25.09, 35.94)	31.29 (26.76, 35.83)	
College graduate or above	19.88 (16.99,22.76)	22.67 (17.60, 27.73)	22.77 (17.49, 28.05)	18.63 (13.77, 23.48)	15.12 (10.96, 19.28)	
PIR,%						0.50
< 1	15.49 (13.62,17.36)	17.07 (13.47, 20.66)	13.95 (10.74, 17.15)	16.61 (13.03, 20.20)	19.10 (15.06, 23.14)	
1–4	48.18 (43.35,53.02)	51.62 (45.62, 57.61)	54.76 (49.01,60.51)	48.68 (42.73, 54.63)	51.18 (45.46, 56.89)	
> 4	29.68 (25.61,33.76)	31.32 (25.07, 37.57)	31.29 (25.22, 37.36)	34.70 (28.56, 40.85)	29.73 (23.35, 36.10)	
BMI, %						** < 0.0001**
Normal weight	11.18 (9.12, 13.24)	18.21 (13.32, 23.09)	11.21 (8.48, 13.93)	8.57 ( 5.54, 11.60)	7.87 (5.14, 10.60)	
Overweight	23.61 (20.91,26.30)	36.12 (1.85)	38.85 (1.93)	35.94 (1.74)	32.97 (1.47)	
Obesity	63.13 (57.53,68.71)	57.17 (51.61, 62.72)	63.08 (57.50, 68.67)	65.85 (60.75, 70.95)	71.56 (66.72,76.40)	
Smoke, %						0.11
Never	49.35 (45.30,53.40)	54.70 (49.43, 59.97)	51.86 (46.77, 56.96)	45.37 (39.47, 51.26)	45.42 (40.12, 50.73)	
Former	27.21 (24.01,30.41)	24.18 (19.36,29.01)	25.84 (21.00, 30.67)	31.20 (25.70, 36.70)	27.49 (22.92,32.06)	
Now	23.44 (20.49,26.40)	21.12 (16.87, 25.36)	22.30 (18.03, 26.57)	23.44 (18.87, 28.00)	27.09 (22.99, 31.18)	
Alcohol use, %	72.54 (67.29,77.78)	77.05 (73.13, 80.98)	73.42 (68.90, 77.94)	71.52 (67.37, 75.66)	68.09 (63.31,72.87)	**0.04**
Hypertension, %	62.86 (57.44,68.27)	59.54 (53.54, 65.54)	61.73 (56.01, 67.45)	64.10 (58.55, 69.64)	65.98 (60.42, 71.55)	** < 0.0001**

LDL-C, Low-density lipoprotein cholesterol; HDL-C, High-density lipoprotein cholesterol; BMI, Body mass index; PIR, Family income-poverty ratio; DM: diabetes

Bold value indicates statistical significance

### Association of TyG index with all-cause mortality and CVD mortality

Table 2 illustrates the incidence of 332 all-cause deaths and 88 CVD-related deaths during a follow-up period of 8.2 years. Three Cox regression models were formulated to explore the independent association between the TyG index and all-cause mortality and CVD mortality risk. In Model 1, the TyG index was significantly positive with the risk of all-cause mortality (HR 1.28, 95% CI 1.08–1.42). This positive association persisted in the minimally adjusted model (HR 1.28, 95% CI 1.10–1.50). After full adjustment, the TyG index remained positively linked to the risk of all-cause mortality (HR 1.43, 95% CI 1.12–1.83). Similar results were shown when we categorized participants into quartiles by the TyG index. In Model 1 and Model 2, compared with those in the lowest quartile of the TyG index, individuals in the highest TyG index quartile had a higher risk of all-cause mortality (Model 1: 1.46, 95% CI1.02–2.09; Model 2: 1.53, 95% CI 1.05–2.24). In the fully adjusted Model 3, the HRs (95% CI) for all-cause mortality risk for quartile 2, quartile 3, and quartile 4 of the TyG index were 0.62 (0.35–1.11), 1.10 (0.67–1.81), 1.60 (1.01–2.54), respectively.

**Table 2:** HRs (95% CI) for mortality according to the TyG index

	HR (95% CI) *P* value		
	Model 1	Model 2	Model 3
All-cause mortality			
TyG index (continuous)	1.24 (1.08, 1.42) **0.002**	1.28 (1.10, 1.50) **0.001**	1.43 (1.12, 1.83) **0.004**
TyG index (quartiles)			
Quartile 1	Reference	Reference	Reference
Quartile 2	0.64 (0.39, 1.05) 0.64	0.58 (0.35, 0.96) **0.03**	0.62 (0.35, 1.11) 0.11
Quartile 3	1.09 (1.01, 1.65)** 0.04**	1.22 (1.02, 1.58) **0.03**	1.10 (0.67, 1.81) 0.70
Quartile 4	1.46 (1.02, 2.09) **0.04**	1.53 (1.05, 2.24) **0.03**	1.60 (1.01, 2.54) **0.04**
CVD mortality			
TyG index (continuous)	1.19 (1.01, 1.39) 0.04	1.35 (1.05, 1.74) 0.02	1.61 ( 1.08, 3.05) 0.04
TyG index (quartiles)			
Quartile 1	Reference	Reference	Reference
Quartile 2	0.54 (0.22, 1.32) 0.18	0.48 (0.18, 1.26) 0.14	0.45 (0.16, 1.23) 0.12
Quartile 3	1.39 (1.04, 2.14) 0.02	1.07 (1.04, 1.11) 0.04	1.06 (1.03, 1.09) 0.01
Quartile 4	1.75 (1.07, 3.22) 0.02	1.66 (1.08, 3.40) 0.01	1.69 (1.22, 4.27) 0.01

Model 1: No covariates were adjusted

Model 2: Age, gender, and race were adjusted

Model 3: Age, gender, race, educational levels, PIR, BMI, hypertension, HDL-C, LDL-C, alcohol use, and smoking status were adjusted

HR, Hazard ratio; 95% CI, 95% Confidence Interval

Bold value indicates statistical significance

We observed a positive association between a higher TyG index and an elevated risk of CVD mortality. This association was statistically significant in both Model 1 (HR 1.19, 95% CI 1.01–1.39) and Model 2 (HR 1.35, 95% CI 1.05–1.74). Furthermore, this positive association between the TyG index and the risk of CVD mortality remained robust in Model 3 (HR 1.61, 95% CI 1.08–3.05). When the TyG index was categorized into quartiles, the multivariable-adjusted HRs (95% CI) from the lowest quartile to the highest quartile of the TyG index for CVD mortality was 1.00 (reference), 0.45 (0.16–1.23), 1.06 (1.03–1.09), 1.69 (1.22–4.27) respectively.

### RCS analysis

**Fig. 2:**
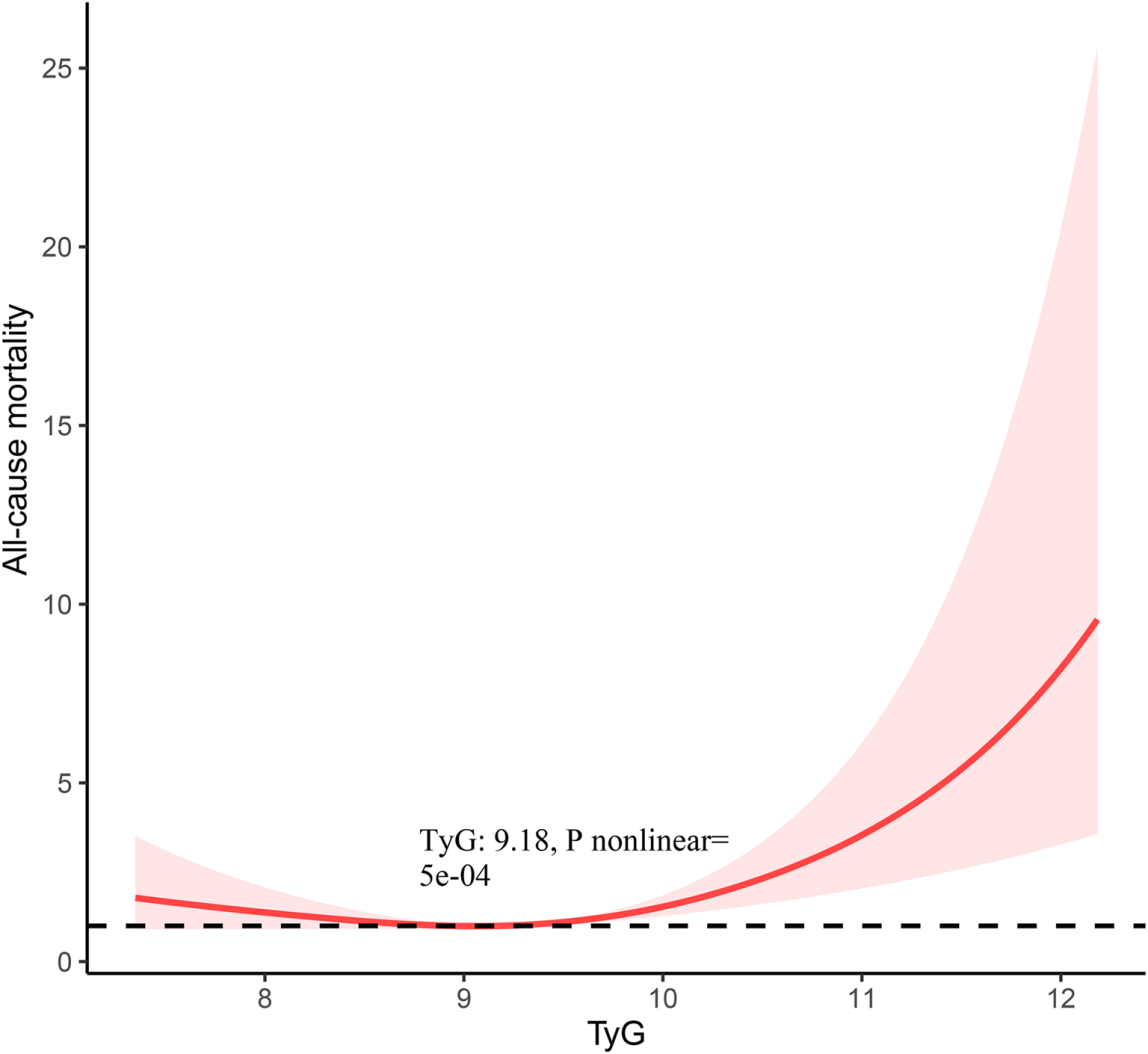
The Restricted cubic spline (RCS) analysis between the TyG index and the all-cause mortality

**Fig. 3:**
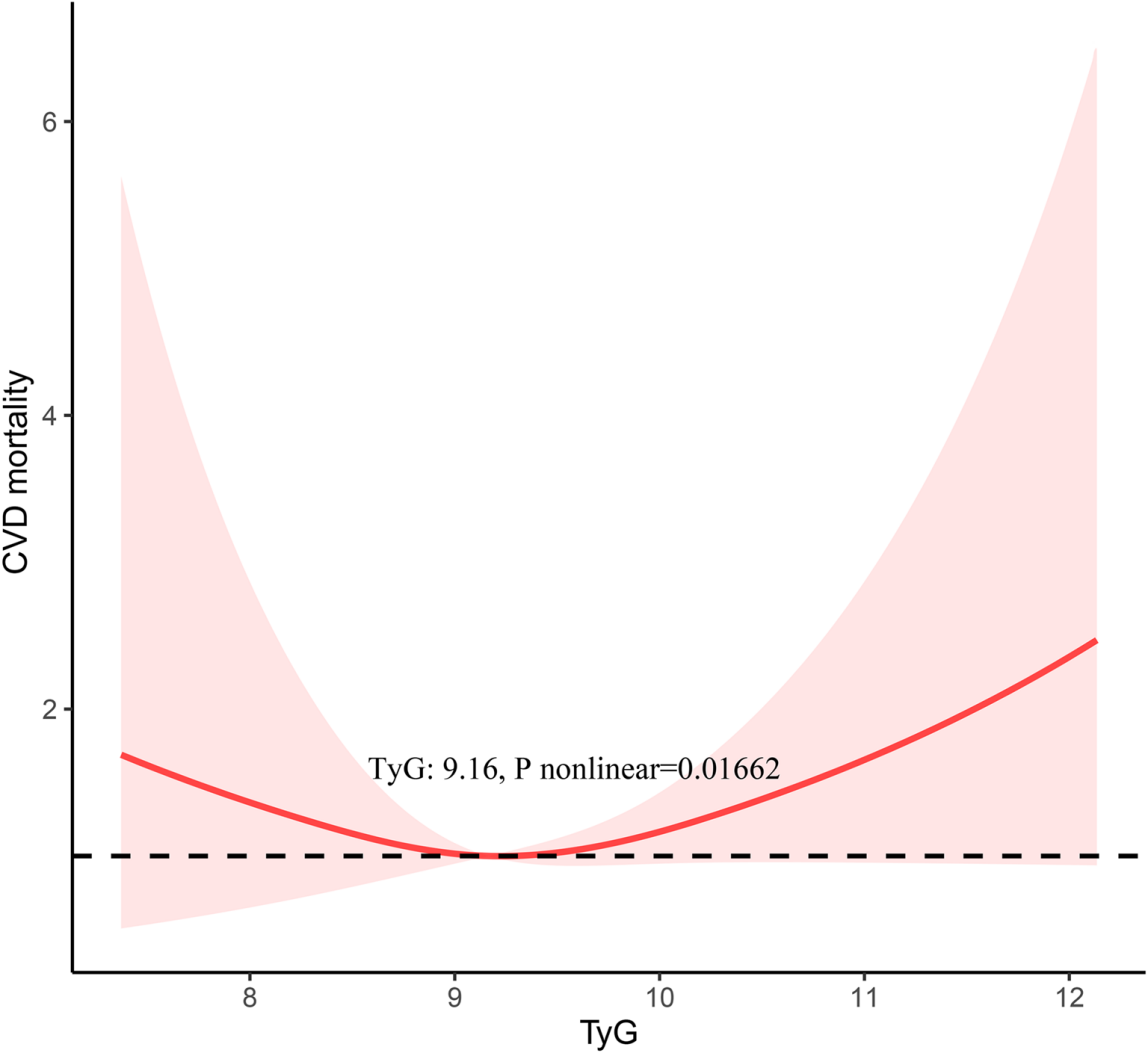
The restricted cubic spline (RCS) analysis between the TyG index and the CVD mortality

In our multivariate Cox regression analysis, we identified a possible nonlinear association between the TyG index and all-cause mortality and CVD mortality. To further validate the association between the TyG index and the risk of all-cause mortality and CVD mortality, we employed RCS analysis. Our results demonstrated a U-shaped association between the TyG index and all-cause mortality and CVD mortality (Figs. 2 and 3). In addition to this, we further fitted the association between baseline TyG index and mortality using two segmented Cox proportional hazards regression models. Remarkably, we identified threshold values of 9.18 for all-cause mortality and 9.16 for CVD mortality (Table 3). Notably, when the TyG index was ≥ 9.18, we observed that a 1-unit increase in the TyG index was associated with a 77% increase in the risk of all-cause mortality (HR 1.77, 95% CI 1.05–2.96). When the TyG index was < 9.18, the risk of all-cause mortality was not significantly associated with changes in the TyG index. The risk of CVD mortality decreased at the lowest level when each unit of the TyG index increased to a critical value. And when the TyG index was ≥ 9.16, it showed a significant positive association with the risk of CVD mortality (HR 2.38, 95% CI 1.05–5.38).

**Table 3:** Threshold effect analysis of TyG index on all-cause and CVD mortality in patients aged ≤ 65 with diabetes

	HR (95% CI) *P* value
All-cause mortality	
Fitting by two-piecewise Cox proportional risk model	
Inflection point	9.18
TyG index < 9.18	0.75 (0.36, 1.58) 0.45
TyG index ≥ 9.18	1.77 (1.05, 2.96) **0.03**
*P* for log-likelihood ratio	** < 0.001**
CVD mortality	
Fitting by two-piecewise Cox proportional risk model	
Inflection point	9.16
TyG index < 9.16	0.60 (0.23, 1.59) 0.30
TyG index ≥ 9.16	2.38 (1.05, 5.38) **0.04**
*P* for log-likelihood ratio	** < 0.001**

HR, Hazard ratio; 95% CI, 95% Confidence Interval

Bold value indicates statistical significance

### Subgroup analysis

To further validate the high TyG index group (≥ 9.16 or 9.18) and the low TyG index group (< 9.16 or 9.18), we performed subgroup analyses (Tables 4 and 5). The analyses were stratified by gender, BMI, hypertension, smoking, and drinking status. Our results revealed a positive association between the TyG index and the risk of all-cause mortality among males (HR 2.19, 95% CI 1.14–3.83), individuals classified as overweight (HR 1.67, 95% CI 1.67–10.18), those with hypertension (HR 2.11, 95% CI 1.25–3.53), current smokers (HR 3.01, 95% CI 1.41–6.43), and alcohol consumers (HR 2.63, 95% CI 1.52–4.54) when the TyG index exceeded the threshold of 9.18. Notably, no significant interactions were observed between the TyG index and the stratification variables.

**Table 4:** Stratified analyses of the associations between TyG index and all-cause mortality

		HR (95% CI) *P* value	
All-cause mortality			
STyG index	< 9.18	> 9.18	P for interaction
Gender			0.90
Female	Ref	1.66 (0.89, 3.11) 0.11	
Male	Ref	2.19 (1.14, 3.83) 0.02	
BMI			
Normal weight	Ref	1.54 (0.53, 4.47) 0.42	0.06
Overweight	Ref	4.12 (1.67, 10.18) 0.003	
Obesity	Ref	1.50 (0.84, 2.67) 0.16	
Hypertension			0.53
Yes	Ref	2.11 (1.25, 3.53) 0.01	
No	Ref	1.93 (0.80, 4.61) 0.14	
Smoke			0.14
Never	Ref	1.84 (0.86, 3.94) 0.11	
Former	Ref	1.20 (0.54, 2.65) 0.65	
Now	Ref	3.01 (1.41, 6.43) 0.01	
Alcohol use			0.16
Yes	Ref	2.63 (1.52, 4.54) < 0.001	
No	Ref	1.17 (0.56, 2.45) 0.66	

HR, Hazard ratio; 95% CI, 95% Confidence Interval

**Table 5:** Stratified analyses of the associations between TyG index and CVD mortality

		HR (95% CI) *P*-value	
CVD mortality			
TyG index	< 9.16	> 9.16	P for interaction
Gender			0.92
Female	Ref	2.18 (0.58, 8.16) 0.24	
Male	Ref	2.54 (1.14, 5.64) 0.02	
BMI			0.40
Normal weight	Ref	0.63 (0.06, 6.18) 0.67	
Overweight	Ref	3.40 (0.74, 15.55) 0.11	
Obesity	Ref	2.14 (0.85, 5.40) 0.11	
Hypertension			0.54
Yes	Ref	2.55 (1.07, 6.09) 0.04	
No	Ref	1.13 (0.27, 4.80) 0.86	
Smoke			0.49
Never	Ref	2.54 (0.57, 11.25) 0.22	
Former	Ref	1.72 (0.41, 7.11) 0.45	
Now	Ref	2.85 (1.01, 8.13) 0.04	
Alcohol use			0.56
Yes	Ref	1.74 (0.61, 4.95) 0.29	
No	Ref	3.69 (1.23, 11.01) 0.02	

HR, Hazard ratio; 95% CI, 95% Confidence interval

In contrast, when the TyG index was above the threshold of 9.16, we found a significant increase in CVD mortality among men (HR 2.54, 95% CI 1.14–5.64), hypertensive patients (HR 2.55, 95% CI 1.07–6.09), current smokers (HR 2.85, 95% CI 1.01–8.13), and nondrinkers (HR 3.69, 95% CI 1.23–11.01). Additionally, interaction tests indicated no significant differences among the various strata, illustrating the lack of a significant effect of the stratification variables on this positive association (all *P* for interaction > 0.05).

## Discussion

In this current study involving 2440 young US patients with diabetes, we observed a U-shaped association between TyG index and all-cause and CVD mortality. In addition to this, threshold effect analysis revealed the inflection point of the TyG index with all-cause mortality (9.18) and CVD mortality (9.16). The results of subgroup analyses revealed no significant interactions observed between the TyG index and stratification variables. In conclusion, our study suggests that the TyG index is a valuable marker of risk of all-cause mortality and CVD mortality in young patients with diabetes, which may help to advance CVD prevention measures.

The TyG index has clear advantages over other indices as a valuable index for assessing the potential for IR [8]. Its association with atherosclerosis and coronary artery calcification underscores its close association with CVD [21, 22]. Previous studies have demonstrated the association between TyG index and the incidence and mortality of CVD disease in different populations. For instance, a study revealed a notable connection between elevated TyG indices and heightened incidences of coronary artery disease and myocardial infarction within the general population [12]. In patients with stable cardiovascular disease, there was a positive association between TyG index and the occurrence of future adverse cardiovascular events such as nonfatal myocardial infarction, stroke, and postdischarge revascularization [23]. Wu et al. identified the TyG index as a predictive factor for major adverse cardiovascular events among patients with premature coronary artery disease [24]. Moreover, an increased TyG index correlates with a higher risk of cardiovascular mortality among individuals deemed at elevated risk of CVD [13]. TyG index can also be a simple and convenient marker for early identification of future CVD disease in postmenopausal women [25]. In our study, we have identified a nonlinear association between the TyG index and the risk of CVD mortality. Specifically, we observed a positive association with CVD mortality when the TyG index exceeded 9.16. Similar to our findings, one study found a positive association between TyG index and CVD mortality when it exceeded 9.52 [26]. Zhao et al. also found a positive association with CVD mortality when TyG index exceeded 9 [27]. Furthermore, it is noteworthy that certain studies have indicated a reduced risk of CVD mortality when the baseline TyG index falls below a specific threshold [28]. In addition to this, studies have also found an increased incidence of diabetic nephropathy with either a TyG index below or above the threshold [29]. Low triglyceride levels have been identified as an adverse prognostic indicator for cardiovascular mortality in individuals with chronic heart failure [30]. Similarly, in patients experiencing stroke unrelated to cardiac embolism, decreased serum triglyceride levels emerged as an independent predictor of mortality following ischemic stroke [31]. Our results also found an association with an increased risk of all-cause mortality when the TyG index was greater than 9.18. This is consistent with the results of several previous studies. Sun et al. found that an elevated TyG index was associated with an increased risk of all-cause mortality in American men over 45 years of age [32]. Shen et al. also found that a higher TyG index was associated with an increased risk of all-cause mortality in diabetic patients with acute coronary syndromes [33]. There are also some studies that are not consistent with our results. Lopez-Jaramillo et al. did not find a significant association between TyG index and non-cardiovascular mortality and all-cause mortality [34]. No association between TyG index and all-cause mortality was also reported by Liu et al. [12]. These findings may vary depending on factors such as age, heterogeneity of the patient population, etc. Inconsistencies in the results of the various studies may also be due to differences in geographic location and economic status [35].

The mechanism by which TyG indices are associated with increased mortality is currently unknown, but IR may hold a key position between the association between TyG and mortality. IR is a pathophysiological disorder that manifests itself primarily in the form of decreased insulin sensitivity of muscle and liver tissues, resulting in a reduced bioavailability of insulin, a diminished capacity to uptake glucose, and ultimately hyperglycemia [36]. The hyperglycemic state further induces inflammation, as evidenced by elevated inflammatory cytokines such as tumor necrosis factor alpha (TNF-α), interleukin (IL)-6, and IL-8, the production of which further leads to smooth muscle cell proliferation and collagen deposition, ultimately leading to vascular senescence and vascular sclerosis [37–39]. IR also inhibits the expression of adiponectin, which has been reported to increase the risk of coronary artery disease in male patients with hypoadiponectinemia [40]. IR also promotes the production of fibrinogen activator inhibitor-1, which further contributes to thrombosis [41], in addition to elevated expression of adhesion-inducing factor and thromboxane A2-dependent tissue factor in platelets, which further promotes platelet activation [42]. IR-induced systemic dyslipidemia may also contribute to the development of atherosclerosis [43]. IR is also associated with enhanced oxidative stress, and excessive levels of oxidative stress are thought to be a key factor in stimulating smooth muscle cell migration and collagen deposition into damaged endothelial cell sites [44–48]. Inadequate bioavailability of nitric oxide in the endothelium further contributes to impaired endothelial function and greater endothelial cell damage [49, 50].

This study has several strengths. First, the study population for this study was drawn from a nationally representative NHANES population identified by a complex multistage probability sampling methodology and included 2440 young adults with diabetes, a relatively rich sample size. Second, our study evaluated the association between the TyG index for all-cause and CVD mortality in young patients with diabetes, enriching the research in this area. Third, this study used stratified and sensitivity analyses to further ensure the reliability and robustness of the findings.

This study also has some limitations that cannot be ignored. Firstly, our population in this study was predominantly young Americans, which may result in limited extrapolation of findings due to differences in ethnicity and life circumstances, and further validation of the generalizability of our main findings to populations of different ethnicities and broader age ranges is needed. Secondly, although we have adjusted for potential confounders, we have not been able to completely rule out all potential confounding bargains, and we also lacked an assessment of all metabolic indices associated with IR, e.g., due to a lack of fasting insulin levels. Thirdly, with only a singular baseline value for the TyG index, the impact of fluctuations in this index during follow-up on mortality risk remains ambiguous. Last but not least, because the NHANES design data did not clarify type 1 or type 2 diabetes in its study design, we could not further evaluate this association for subgroups stratified by type 1 and type 2 diabetes. Finally, our findings are observational, and the causal role of the TyG index on all-cause mortality and CVD mortality should be validated in further prospective intervention studies. Despite these limitations, our study contributes to the existing knowledge base, and future research with more extensive data collection could further elucidate these relationships.

## Conclusion

Our findings suggest that the TyG index is an economical and valuable biomarker of the risk of all-cause mortality and CVD mortality in young patients with diabetes under 65 years of age in the United States. We also found a nonlinear association between the TyG index and all-cause and CVD mortality. Therefore, we advocate for the monitoring of the TyG index in young patients with diabetes as a potentially beneficial approach for assessing clinical prognosis and mortality risk in these patients.

## Data Availability

Publicly available datasets were analyzed in this study. This data can be found here: https://www.cdc.gov/nchs/nhanes/index.htm.
